# Screening for ATTR amyloidosis in the clinic: overlapping disorders, misdiagnosis, and multiorgan awareness

**DOI:** 10.1007/s10741-021-10080-2

**Published:** 2021-02-20

**Authors:** Jose N. Nativi-Nicolau, Chafic Karam, Sami Khella, Mathew S. Maurer

**Affiliations:** 1 Department of Internal Medicine, University of Utah Health, Salt Lake City, UT USA; 2 Department of Neurology, Oregon Health & Science University, Portland, OR USA; 3 Department of Neurology, University of Pennsylvania, Philadelphia, PA USA; 4 Department of Medicine, Columbia University, New York, NY USA

**Keywords:** Amyloidosis, ATTRv, hATTR, Cardiomyopathy, Transthyretin amyloidosis

## Abstract

Amyloid transthyretin (ATTR) amyloidosis is a clinically heterogeneous and fatal disease that results from deposition of insoluble amyloid fibrils in various organs and tissues, causing progressive loss of function. The objective of this review is to increase awareness and diagnosis of ATTR amyloidosis by improving recognition of its overlapping conditions, misdiagnosis, and multiorgan presentation. Cardiac manifestations include heart failure, atrial fibrillation, intolerance to previously prescribed antihypertensives, sinus node dysfunction, and atrioventricular block, resulting in the need for permanent pacing. Neurologic manifestations include progressive sensorimotor neuropathy (e.g., pain, weakness) and autonomic dysfunction (e.g., erectile dysfunction, chronic diarrhea, orthostatic hypotension). Non-cardiac red flags often precede the diagnosis of ATTR amyloidosis and include musculoskeletal manifestations (e.g., carpal tunnel syndrome, lumbar spinal stenosis, spontaneous rupture of the distal tendon biceps, shoulder and knee surgery). Awareness and recognition of the constellation of symptoms, including cardiac, neurologic, and musculoskeletal manifestations, will help with early diagnosis of ATTR amyloidosis and faster access to therapies, thereby slowing the progression of this debilitating disease.

## Introduction

Amyloid transthyretin (ATTR) amyloidosis is a progressively debilitating, clinically heterogeneous, and fatal disease caused by the buildup of transthyretin (TTR) amyloid fibrils in various organs and tissues, resulting in multisystem dysfunction particularly in the heart, along with the peripheral and autonomic nervous systems [1–3]. The diagnosis of ATTR amyloidosis has been increasing over the last decade, and many patients have had musculoskeletal manifestations, such as carpal tunnel syndrome, distal biceps tendon rupture, idiopathic trigger finger, or spinal stenosis, any or all of which can precede by several years the cardiac or neurologic manifestations [3–8]. Disease progression in patients with ATTR amyloidosis is remarkably fast, resulting in significant impairment of function and irretrievable loss of quality of life [9–13]. With therapies available to slow disease progression, early recognition and diagnosis of patients with ATTR amyloidosis are important to facilitate early treatment. The aim of this review is to increase awareness of the constellation of symptoms in patients with ATTR amyloidosis—especially the non-cardiac symptoms that cardiologists and others may not traditionally associate with ATTR amyloidosis but that are key for identifying patients with this progressive, fatal disease.

## Wild-type vs hereditary ATTR amyloidosis symptoms

There are two forms of ATTR amyloidosis: wild type (ATTRwt) and hereditary (ATTRv [variant]). In ATTRwt amyloidosis, which was previously termed senile cardiac amyloidosis, a native non-mutated TTR protein misfolds into amyloid fibrils, primarily resulting in dysfunction of the heart that is characterized by restrictive cardiomyopathy; this is predominantly seen in males aged > 60 years [14–16]. Although ATTRwt amyloidosis typically manifests as cardiac symptoms, patients may also have signs and symptoms of sensorimotor neuropathy and autonomic neuropathy [14, 15], along with a clinical history of carpal tunnel syndrome, spinal stenosis, and other musculoskeletal manifestations [7]. ATTRv amyloidosis, originally called familial amyloidotic polyneuropathy, is caused by a single amino acid substitution produced by a point mutation in the *TTR* gene. More than 130 mutations have been identified to date, with some mutations more often associated with either predominant polyneuropathy or cardiomyopathy; however, most patients experience a mixed phenotype with both neuropathic and cardiac symptoms [14, 15, 17–19]. The mechanisms by which mutations influence TTR aggregation or fibril morphology leading to organ dysfunction with such variable clinical presentations are poorly understood [20, 21]. In addition, phenotypic expression can be highly variable among individuals with a specific mutation, even within the same family [14].

## Overlapping conditions and misdiagnosis of ATTR amyloidosis

ATTR amyloidosis is often overlooked or misdiagnosed in patients, at least early in its course, due to the non-specific, heterogeneous, multisystem presentation of the disease [3]. As the disease progresses, the symptoms and clinical manifestations of ATTR amyloidosis often mimic those of other more common diseases, further complicating and delaying diagnosis [22–24]. Thus, patients with ATTR amyloidosis could receive inappropriate treatments, such as chemotherapy for light-chain amyloidosis and intravenous immunoglobulins or steroids for immune polyneuropathies [3, 25, 26].

The signs and symptoms that should raise suspicion of ATTR amyloidosis with cardiomyopathy (ATTR-CM) often overlap with other more commonly recognized cardiovascular diseases, such as heart failure with preserved ejection fraction, hypertensive cardiomyopathy, aortic stenosis, hypertrophic cardiomyopathy, and light chain amyloidosis (Table 1) [25, 27–29]. Given that the life expectancy of a patient with ATTR-CM is 2 to 5 years after diagnosis, early and accurate diagnosis is key to forestalling disease progression. Recognizing the disease’s signs and symptoms, which affect multiple systems, may aid cardiologists in avoiding misdiagnosis.

**Table 1:** Overlapping conditions and misdiagnosis of ATTR amyloidosis

Cardiac [27–29]	Neurologic [3, 24, 25]
• Heart failure with preserved ejection fraction • Hypertensive cardiomyopathy • Aortic stenosis • Hypertrophic cardiomyopathy • Light chain amyloidosis with cardiac involvement • Idiopathic restrictive cardiomyopathy • Iron overload • Other infiltrative cardiomyopathies (e.g., Fabry disease)	• Chronic inflammatory demyelinating polyneuropathy • Paraproteinemic peripheral neuropathy (e.g., monoclonal gammopathy-associated) • Toxic peripheral neuropathy • Vasculitic peripheral neuropathy • Idiopathic axonal polyneuropathy • Paraneoplastic neuropathy • Diabetic neuropathy • Alcoholic neuropathy • Motor neuron disease (e.g., amyotrophic lateral sclerosis) • Fibromyalgia • Light chain amyloidosis

*ATTR* amyloid transthyretin

## Recognizing a constellation of ATTR amyloidosis symptoms

**Fig. 1:**
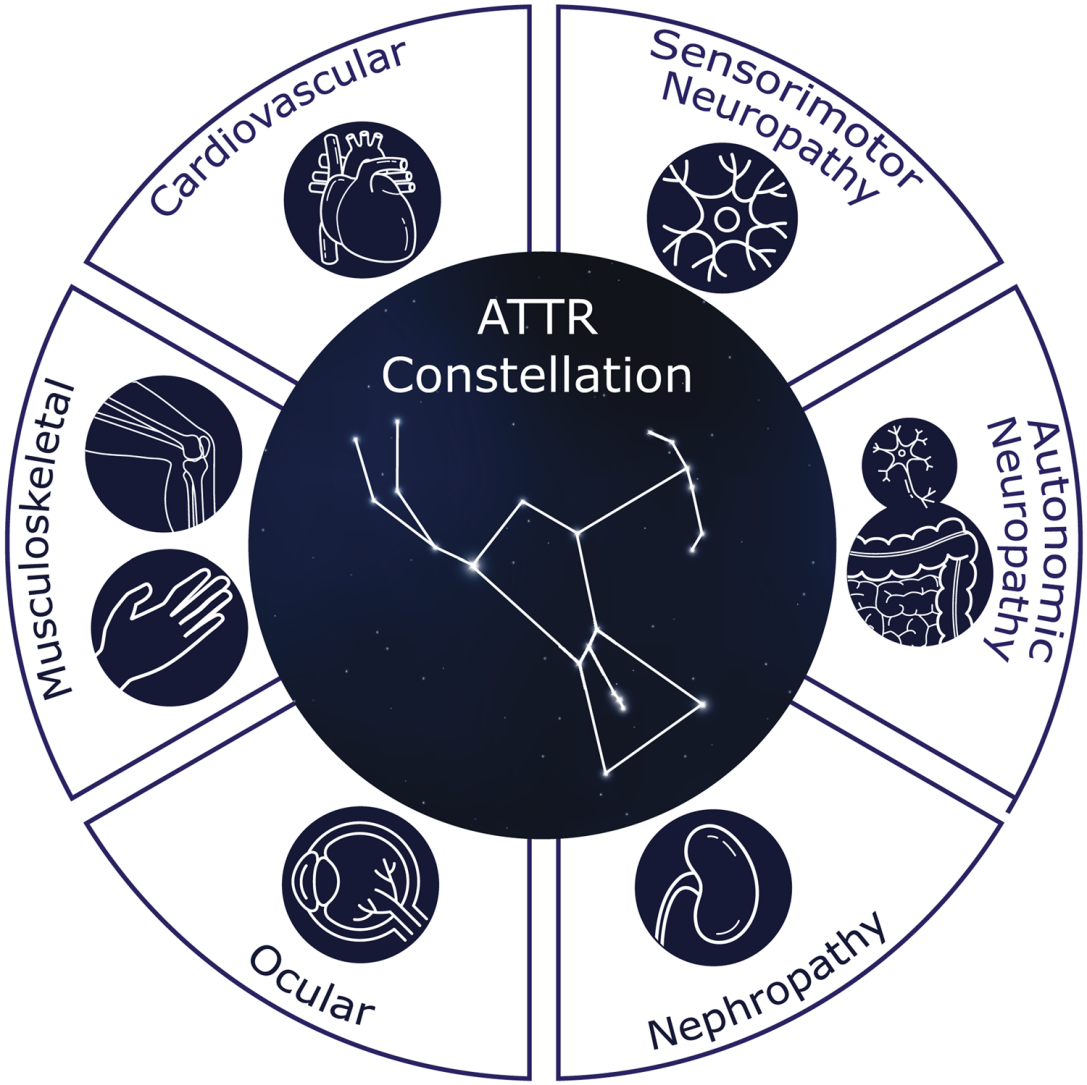
A constellation of multisystem clinical signs and symptoms increases awareness of amyloid transthyretin (ATTR) amyloidosis. Recognition of non-cardiac symptoms clustered with cardiac and/or neurologic symptoms should prompt diagnostic testing and patient referral to a multidisciplinary team at an amyloidosis expert center

**Fig. 2:**
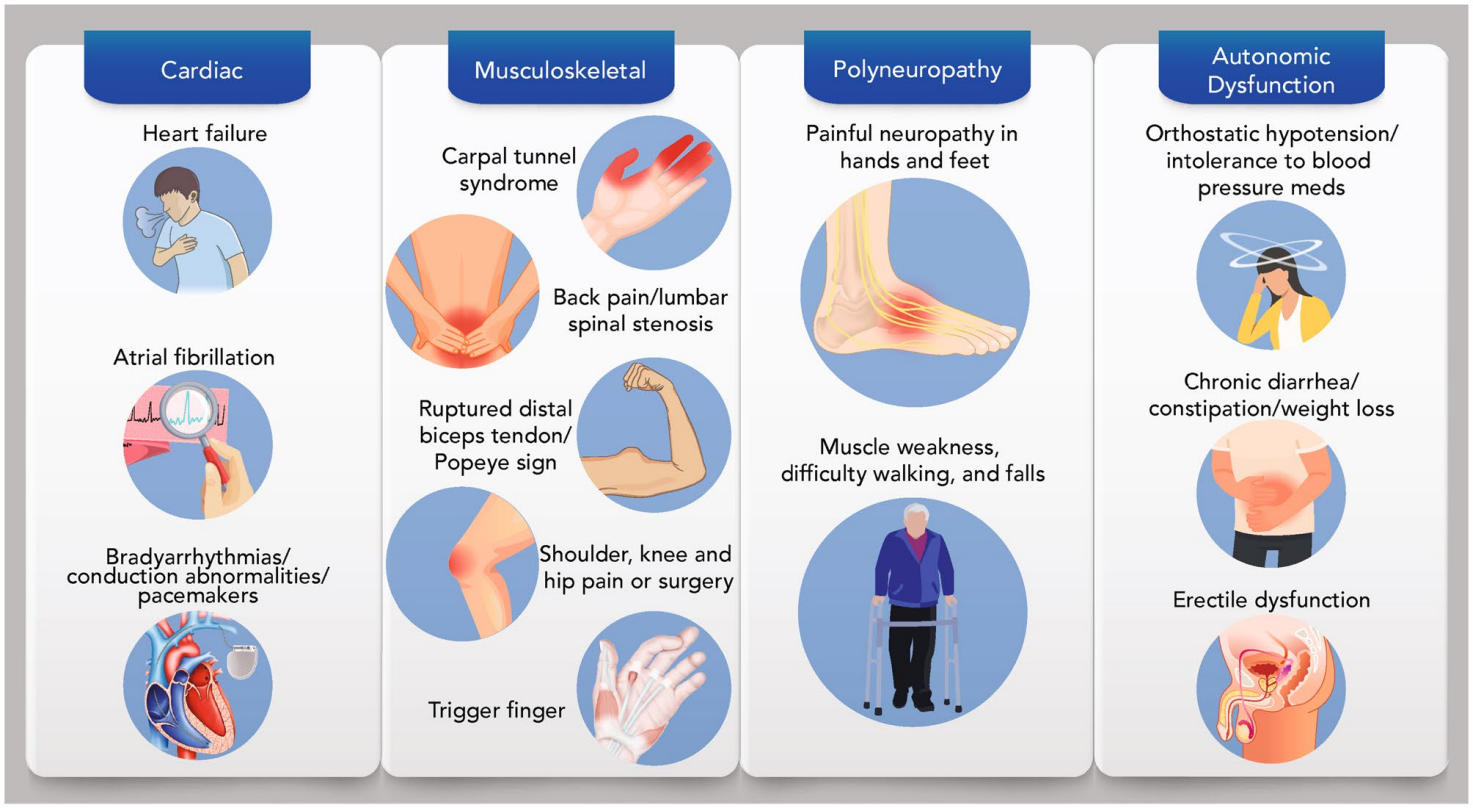
Symptoms of ATTR amyloidosis. Patients with ATTR amyloidosis may present with clinical signs or symptoms of cardiomyopathy or progressive polyneuropathy along with musculoskeletal symptoms and signs of autonomic dysfunction. ATTR amyloidosis should be considered for patients with cardiac, neurologic, or musculoskeletal manifestations, particularly when those symptoms suggest multiple organs are affected. *ATTR* amyloid transthyretin

Early suspicion and recognition of ATTR amyloidosis can lead to an earlier diagnosis and treatment; there is evidence to suggest that a delay in treatment leads to irretrievable loss of quality of life and progression of the polyneuropathic and cardiac manifestations for most patients [3, 9–13]. Recognition of a constellation of symptoms may raise suspicion of amyloidosis early in its course (Fig. 1). Although patients may present with predominant symptoms of cardiomyopathy or progressive polyneuropathy, there can be substantial overlap, with many individuals presenting with a combination of both, as well as other abnormalities, such as musculoskeletal symptoms, orthostatic hypotension, erectile dysfunction, gastrointestinal abnormalities, and unexplained weight loss (Fig. 2) [14, 15]. Patients may also present with ocular manifestations and symptoms of nephropathy, which are discussed in other reviews [3, 30]. This phenotypic variability poses a considerable diagnostic challenge. ATTR amyloidosis should be considered in patients with signs and symptoms associated with cardiac, neurologic, or musculoskeletal manifestations, particularly when the constellation of those symptoms suggests that multiple organs are affected [3, 31].

## Cardiovascular symptoms of ATTR amyloidosis

ATTR-CM is characterized by increased ventricular wall thickness, increased valve thickness, and interatrial and interventricular septum thickness that present as restrictive cardiomyopathy and progress to heart failure—initially in the setting of preserved ejection fraction, conduction system disturbances, and arrhythmias—with resulting impaired functional capacity, syncope, or palpitations [14, 15, 17, 32–34]. The signs and symptoms that should raise suspicion of ATTR-CM include a history of right-sided heart failure; heart failure with preserved ejection fraction (especially in men); intolerance to angiotensin-converting enzyme inhibitors, angiotensin receptor blockers, angiotensin receptor neprilysin inhibitors (ARNi), or beta-blockers; or atrial arrhythmias, conduction system disease, or need for a pacemaker (Table 2) [28, 29, 35, 36]. Additionally, heart failure in patients with ATTR amyloidosis progressively worsens over time, with patients experiencing decline in diastolic dysfunction, decrease in left ventricular ejection fraction (~3% every 6 months), increased restrictive filling, and decline in functional capacity (~26 m decrease in 6-min walk distance every 6 months) [15, 27, 33, 37–39]. In patients with ATTRwt and ATTRv amyloidosis, troponin levels or N-terminal pro–B-type natriuretic peptide (NT-proBNP) levels are elevated and increase over time; this is an indicator of clinical progression of heart failure [33, 36].

**Table 2:** Signs and symptoms that should raise suspicion of ATTR amyloidosis with cardiomyopathy

Signs/symptoms
Heart failure with predominant right-sided symptoms (e.g., distended jugular veins, anorexia, gastrointestinal upset, dependent edema, weight gain)
HFpEF, especially in men
Intolerance to ACE inhibitors, angiotensin receptor blockers, ARNi, or beta-blockers
Unexplained atrial arrhythmias, conduction system disease, or need for a pacemaker
History of musculoskeletal syndromes or procedures: CTS; lumbar spinal stenosis; spontaneous distal bicep tendon rupture; or shoulder, knee, or hip surgery

*ACE* angiotensin-converting enzyme, *ARNi* angiotensin receptor-neprilysin inhibitors, *ATTR* amyloid transthyretin, *CTS* carpal tunnel syndrome, *HFpEF* heart failure with preserved ejection fraction

## Neurologic symptoms of ATTR amyloidosis

Patients with amyloid polyneuropathy, such as ATTR amyloidosis, are frequently misdiagnosed with chronic inflammatory demyelinating polyradiculoneuropathy (CIDP) [24, 25, 40]. Other neuropathies confused with ATTR amyloidosis include paraproteinemic peripheral neuropathy, toxic peripheral neuropathy, vasculitic peripheral neuropathy, idiopathic axonal polyneuropathy, diabetic polyneuropathy, alcoholic neuropathy, paraneoplastic neuropathy, monoclonal gammopathy–associated neuropathy, and, more rarely, motor neuropathy and amyotrophic lateral sclerosis (Table 1) [24, 25, 40]. Recently published guidelines review in greater detail the misleading features that often lead to these misdiagnoses [24]. Two key features of ATTR amyloidosis with polyneuropathy (ATTR-PN) distinguish it from the more common diabetic polyneuropathy: its progressive nature and, frequently, distal limb weakness. Diabetic polyneuropathy is typically a slow, progressive, and distal sensory neuropathy without much limb weakness (Table 3).

**Table 3:** Comparison of neuropathies related to ATTR amyloidosis and diabetes

Feature	ATTR polyneuropathy	Diabetic polyneuropathy	References
Pain	Mild, moderate, or severe	Mild	[61–64]
Motor weakness	Common	Uncommon	[14, 15, 43, 44, 63, 64]
Muscle loss	Common	Uncommon	[15, 43, 44, 63, 64]
Progression	Months	Years	[44, 62]
Distribution	Distal and occasionally proximal	Distal	[61–64]

*ATTR* amyloid transthyretin

ATTR-PN is characterized by symmetrical length-dependent peripheral neuropathy; depending on the *TTR* mutation in the case of ATTRv amyloidosis, distal and occasionally proximal limb weakness may be prominent [3, 19]. As the disease progresses through each stage, the pattern of progression and class of nerve fiber impacted is reflected through heterogeneous clinical manifestations experienced by the patient with ATTR-PN [19, 41–43]. Early symptoms of ATTR-PN include burning pain, especially in younger patients; older patients experience burning pain, numbness, and loss of pain and temperature sensation, whereas the ability to perceive touch pressure and joint position is relatively preserved [3, 41]. Examination of nerve fiber involvement at this stage demonstrates degeneration of unmyelinated and small myelinated nerve fibers more than large myelinated fibers [41]. As ATTR-PN progresses, muscle weakness increases, especially in the lower limbs; patients with ATTR-PN suffer from progressive lower limb numbness, weakness, and gait imbalance [15, 43–45].

In addition to signs and symptoms of sensorimotor neuropathy, autonomic dysfunction is observed early in the course of ATTR amyloidosis and can precede motor impairment, but it often goes unrecognized [41, 46, 47]. Furthermore, in cases of severe autonomic dysfunction with reduced sympathetic function, the signs and symptoms of heart failure can be masked [48]. Autonomic neuropathy can manifest as orthostatic hypotension, recurrent urinary tract infection, erectile dysfunction, and/or gastrointestinal disturbances [3, 14, 15, 49]. Orthostatic hypotension, which is commonly reported as a symptom of ATTR amyloidosis, may manifest as dizziness or fainting when standing up, blurred vision, confusion, or light-headedness [15, 17, 46]. Meanwhile, gastrointestinal disturbances may include nausea, vomiting, constipation, diarrhea (possibly alternating with constipation), or fecal incontinence and unintentional weight loss [3, 14, 15, 49].

## Musculoskeletal manifestations of ATTR amyloidosis

**Fig. 3:**
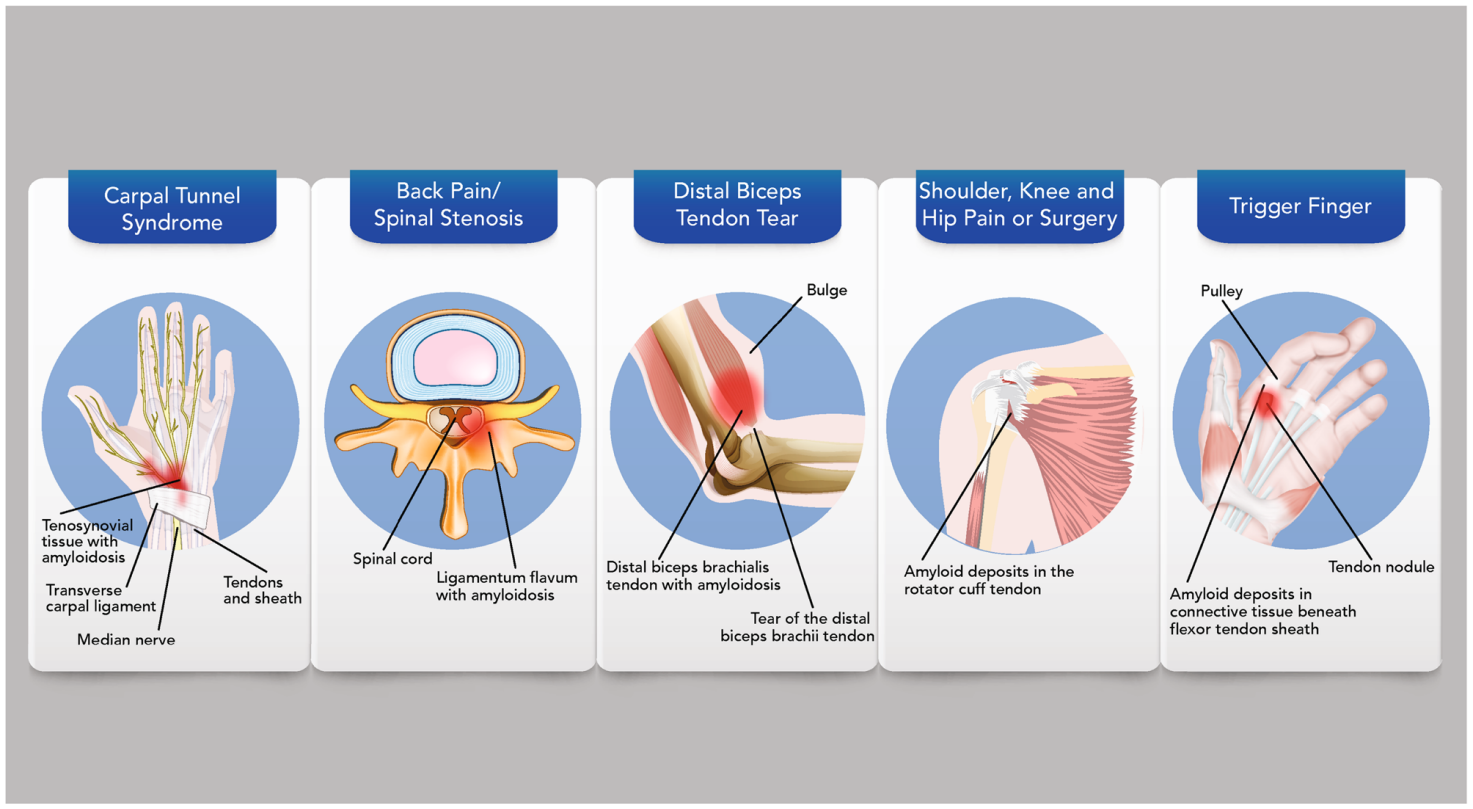
Musculoskeletal manifestations associated with ATTR amyloidosis. Buildup of TTR amyloid fibrils has been detected in tissue-resulting musculoskeletal manifestations, such as carpal tunnel syndrome, spinal stenosis, distal biceps tendon rupture, orthopedic surgery, or idiopathic trigger finger. Patients with ATTR amyloidosis may experience musculoskeletal signs and symptoms years prior to cardiac or neurologic manifestations. *ATTR* amyloid transthyretin; *TTR* transthyretin

Patients with ATTR amyloidosis may develop musculoskeletal manifestations 5 to 15 years prior to other symptoms (Fig. 3) [4, 7, 50, 51]. Numerous studies have reported the presence of ATTR amyloid in tissue removed during orthopedic surgeries, including the flexor tenosynovium, rotator cuff tendons, and ligamentum flavum [52, 53]. Rotator cuff surgery has been predominately reported in patients with ATTRwt amyloidosis [53]. Carpal tunnel syndrome, the most common non-cardiac manifestation in patients with ATTR-CM, often presents years before a diagnosis of ATTRwt or ATTRv amyloidosis [4, 15, 37, 51, 54]. Carpal tunnel syndrome is caused by median nerve compression resulting in numbness, tingling sensations, or hand weakness. A recent study found that 10.2% of patients with bilateral carpal tunnel syndrome tested positive for amyloid deposits [6]. Of the 10 patients identified in the study, five were diagnosed with ATTRwt amyloidosis and two with ATTRv amyloidosis, and several also had a clinical history of trigger finger, lumbar spinal stenosis, or biceps tendon rupture [6]. Trigger finger due to amyloidosis is thought to occur when amyloid fibrils deposit in connective tissue, causing restricted movement of the flexor tendon, which then results in the finger being stuck in a bent position. The coexistence of trigger finger and carpal tunnel syndrome was also reported in members of a Japanese family with ATTRv amyloidosis [8]. In addition, a clinical history of lumbar spinal stenosis has been reported by several studies in patients with ATTRwt and ATTRv amyloidosis [5, 6, 53, 55]. Similarly, rupture of the distal biceps tendon (also known as Popeye sign) can be an early sign of amyloidosis; in patients aged >50 years, Popeye sign should raise suspicion of ATTR amyloidosis [56].

Orthopedic surgery is significantly more common in patients with ATTR-CM compared with the general population [57]. Arthroplasty typically occurs over 6 to 8 years before diagnosis of ATTR amyloidosis [57]. One study found that 25.9% (28/108) and 18.8% (12/64) of patients with ATTRwt and ATTRv amyloidosis with cardiomyopathy, respectively, underwent hip or knee arthroplasty [57]. In addition, rotator cuff repair occurred in 9.9% of patients with ATTR amyloidosis [53, 57]. A history of a constellation of these musculoskeletal syndromes and surgeries in a patient along with cardiac or neurologic symptoms should raise clinical suspicion and prompt physicians to screen for ATTR amyloidosis [7].

## Implementation of screening for ATTR amyloidosis in clinical practice

Cardiologists should screen for ATTR amyloidosis in patients with clinical signs and symptoms suggestive of multisystem involvement, particularly those with the constellation of cardiac, neurologic, and musculoskeletal manifestations described in this review. Given the multisystemic nature of ATTR amyloidosis, a multidisciplinary approach to assessment, diagnosis, and management of patients is recommended by the guidelines [24, 28]. The assessment of patients with cardiac symptoms should include noninvasive or invasive procedures, as described elsewhere [28, 30, 58, 59].

**Fig. 4:**
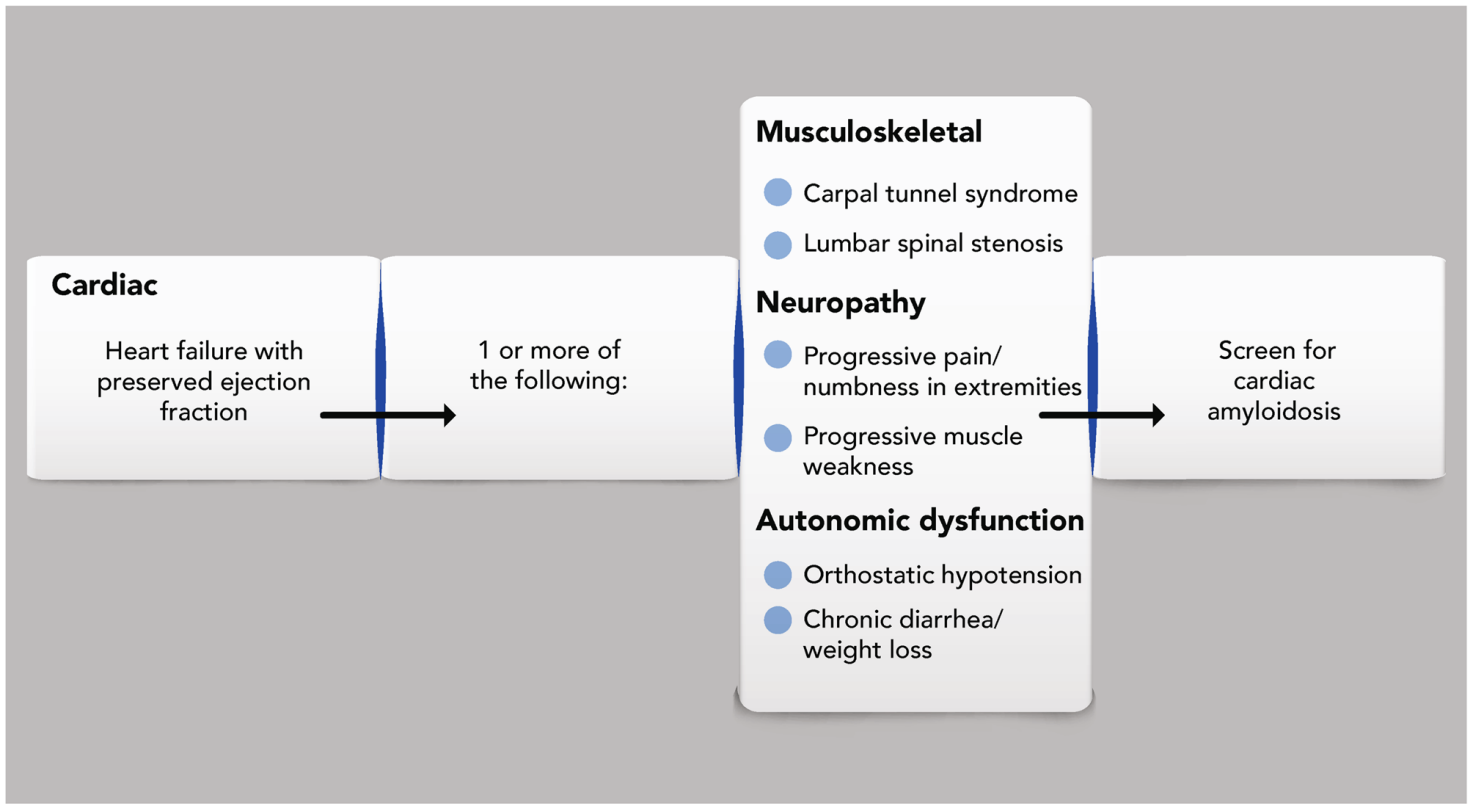
Constellation of symptoms checklist for cardiac ATTR amyloidosis. Healthcare practitioners should evaluate patients with heart failure with preserved ejection fraction for a clinical history of carpal tunnel syndrome or lumbar spinal stenosis, along with progressive neuropathy or autonomic dysfunction. Clustering of these clinical signs and symptoms should prompt screening for cardiac amyloidosis and trigger referral to a multidisciplinary team at an amyloidosis expert center. *ATTR* amyloid transthyretin

It can be challenging to identify ATTR amyloidosis given the diversity of diagnostic clues that can manifest in a patient over time (across many years). As cardiac amyloidosis is present in 10% to 15% of patients with heart failure with preserved ejection fraction [27, 33, 58, 60], the addition of screening questions and a check of multiple symptoms could help to identify patients with ATTR amyloidosis (Fig. 4).

## Conclusion

Awareness of the non-cardiac symptoms that cluster with cardiac and neurologic symptoms can unmask a diagnosis of ATTR amyloidosis and prompt referral to a center with expertise in this disease (Fig. 4) [3, 24, 28]. Because ATTR amyloidosis is now a treatable disease, recognizing the constellation of associated signs and symptoms, including those that are neurologic and musculoskeletal, is important because early treatment will make a meaningful impact on a patient’s quality of life, autonomy, and physical function [9–13].

## References

[1] Costa PP, Figueira AS, Bravo FR (1978). Amyloid fibril protein related to prealbumin in familial amyloidotic polyneuropathy. Proc Natl Acad Sci USA.

[2] Saraiva MJ, Birken S, Costa PP, Goodman DS (1984). Amyloid fibril protein in familial amyloidotic polyneuropathy, Portuguese type. Definition of molecular abnormality in transthyretin (prealbumin). J Clin Invest.

[3] Conceicao I, Gonzalez-Duarte A, Obici L, Schmidt HH, Simoneau D, Ong ML, Amass L (2016). "Red-flag" symptom clusters in transthyretin familial amyloid polyneuropathy. J Peripher Nerv Syst.

[4] Nakagawa M, Sekijima Y, Yazaki M, Tojo K, Yoshinaga T, Doden T, Koyama J, Yanagisawa S, Ikeda S (2016). Carpal tunnel syndrome: a common initial symptom of systemic wild-type ATTR (ATTRwt) amyloidosis. Amyloid.

[5] Ikram A, Donnelly JP, Sperry BW, Samaras C, Valent J, Hanna M (2018). Diflunisal tolerability in transthyretin cardiac amyloidosis: a single center’s experience. Amyloid.

[6] Sperry BW, Reyes BA, Ikram A, Donnelly JP, Phelan D, Jaber WA, Shapiro D, Evans PJ, Maschke S, Kilpatrick SE, Tan CD, Rodriguez ER, Monteiro C, Tang WHW, Kelly JW, Seitz WH, Hanna M (2018). Tenosynovial and cardiac amyloidosis in patients undergoing carpal tunnel release. J Am Coll Cardiol.

[7] Donnelly JP, Hanna M, Sperry BW, Seitz WH (2019). Carpal tunnel syndrome: a potential early, red-flag sign of amyloidosis. J Hand Surg Am.

[8] Uotani K, Kawata A, Nagao M, Mizutani T, Hayashi H (2007). Trigger finger as an initial manifestation of familial amyloid polyneuropathy in a patient with Ile107Val TTR. Intern Med.

[9] Coelho T, Yarlas A, Waddington-Cruz M, White MK, Sikora Kessler A, Lovley A, Pollock M, Guthrie S, Ackermann EJ, Hughes SG, Karam C, Khella S, Gertz M, Merlini G, Obici L, Schmidt HH, Polydefkis M, Dyck PJB, Brannagan Iii TH, Conceicao I, Benson MD, Berk JL (2019). Inotersen preserves or improves quality of life in hereditary transthyretin amyloidosis. J Neurol.

[10] Adams D, Gonzalez-Duarte A, O'Riordan WD, Yang CC, Ueda M, Kristen AV, Tournev I, Schmidt HH, Coelho T, Berk JL, Lin KP, Vita G, Attarian S, Plante-Bordeneuve V, Mezei MM, Campistol JM, Buades J, Brannagan TH, Kim BJ, Oh J, Parman Y, Sekijima Y, Hawkins PN, Solomon SD, Polydefkis M, Dyck PJ, Gandhi PJ, Goyal S, Chen J, Strahs AL, Nochur SV, Sweetser MT, Garg PP, Vaishnaw AK, Gollob JA, Suhr OB (2018). Patisiran, an RNAi therapeutic, for hereditary transthyretin amyloidosis. N Engl J Med.

[11] Benson MD, Waddington-Cruz M, Berk JL, Polydefkis M, Dyck PJ, Wang AK, Plante-Bordeneuve V, Barroso FA, Merlini G, Obici L, Scheinberg M, Brannagan TH, Litchy WJ, Whelan C, Drachman BM, Adams D, Heitner SB, Conceicao I, Schmidt HH, Vita G, Campistol JM, Gamez J, Gorevic PD, Gane E, Shah AM, Solomon SD, Monia BP, Hughes SG, Kwoh TJ, McEvoy BW, Jung SW, Baker BF, Ackermann EJ, Gertz MA, Coelho T (2018). Inotersen treatment for patients with hereditary transthyretin amyloidosis. N Engl J Med.

[12] Maurer MS, Schwartz JH, Gundapaneni B, Elliott PM, Merlini G, Waddington-Cruz M, Kristen AV, Grogan M, Witteles R, Damy T, Drachman BM, Shah SJ, Hanna M, Judge DP, Barsdorf AI, Huber P, Patterson TA, Riley S, Schumacher J, Stewart M, Sultan MB, Rapezzi C (2018). Tafamidis treatment for patients with transthyretin amyloid cardiomyopathy. N Engl J Med.

[13] Obici L, Berk JL, Gonzalez-Duarte A, Coelho T, Gillmore J, Schmidt HH, Schilling M, Yamashita T, Labeyrie C, Brannagan TH, Ajroud-Driss S, Gorevic P, Kristen AV, Franklin J, Chen J, Sweetser MT, Wang JJ, Adams D (2020). Quality of life outcomes in APOLLO, the phase 3 trial of the RNAi therapeutic patisiran in patients with hereditary transthyretin-mediated amyloidosis. Amyloid.

[14] Coelho T, Maurer MS, Suhr OB (2013). THAOS - The Transthyretin Amyloidosis Outcomes Survey: initial report on clinical manifestations in patients with hereditary and wild-type transthyretin amyloidosis. Curr Med Res Opin.

[15] Maurer MS, Hanna M, Grogan M, Dispenzieri A, Witteles R, Drachman B, Judge DP, Lenihan DJ, Gottlieb SS, Shah SJ, Steidley DE, Ventura H, Murali S, Silver MA, Jacoby D, Fedson S, Hummel SL, Kristen AV, Damy T, Plante-Bordeneuve V, Coelho T, Mundayat R, Suhr OB, Waddington CM, Rapezzi C (2016). Genotype and phenotype of transthyretin cardiac amyloidosis: THAOS (Transthyretin Amyloid Outcome Survey). J Am Coll Cardiol.

[16] Ruberg FL, Berk JL (2012). Transthyretin (TTR) cardiac amyloidosis. Circulation.

[17] Rapezzi C, Quarta CC, Obici L, Perfetto F, Longhi S, Salvi F, Biagini E, Lorenzini M, Grigioni F, Leone O, Cappelli F, Palladini G, Rimessi P, Ferlini A, Arpesella G, Pinna AD, Merlini G, Perlini S (2013). Disease profile and differential diagnosis of hereditary transthyretin-related amyloidosis with exclusively cardiac phenotype: an Italian perspective. Eur Heart J.

[18] Rowczenio DM, Noor I, Gillmore JD, Lachmann HJ, Whelan C, Hawkins PN, Obici L, Westermark P, Grateau G, Wechalekar AD (2014). Online registry for mutations in hereditary amyloidosis including nomenclature recommendations. Hum Mutat.

[19] Ando Y, Coelho T, Berk JL, Cruz MW, Ericzon BG, Ikeda S, Lewis WD, Obici L, Plante-Bordeneuve V, Rapezzi C, Said G, Salvi F (2013). Guideline of transthyretin-related hereditary amyloidosis for clinicians. Orphanet J Rare Dis.

[20] Schmidt M, Wiese S, Adak V, Engler J, Agarwal S, Fritz G, Westermark P, Zacharias M, Fandrich M (2019). Cryo-EM structure of a transthyretin-derived amyloid fibril from a patient with hereditary ATTR amyloidosis. Nat Commun.

[21] Schonhoft JD, Monteiro C, Plate L, Eisele YS, Kelly JM, Boland D, Parker CG, Cravatt BF, Teruya S, Helmke S, Maurer M, Berk J, Sekijima Y, Novais M, Coelho T, Powers ET, Kelly JW (2017) Peptide probes detect misfolded transthyretin oligomers in plasma of hereditary amyloidosis patients. Sci Transl Med 9:eaam7621 10.1126/scitranslmed.aam7621 PMC5628019 28904227

[22] Lane T, Bangova A, Fontana M, Hutt DF, Strehina SG, Whelan CJ, Hawkins PN (2015). Quality of life in ATTR amyloidosis. Orphanet J Rare Dis.

[23] Lousada I, Comenzo RL, Landau H, Guthrie S, Merlini G (2015). Patient experience with hereditary and senile systemic amyloidoses: a survey from the Amyloidosis Research Consortium. Orphanet J Rare Dis.

[24] Adams D, Ando Y, Beirao JM, Coelho T, Gertz MA, Gillmore JD, Hawkins PN, Lousada I, Suhr OB, Merlini G (2020) Expert consensus recommendations to improve diagnosis of ATTR amyloidosis with polyneuropathy. J Neurol. Online ahead of print 10.1007/s00415-019-09688-0 PMC8179912 31907599

[25] Cortese A, Vegezzi E, Lozza A, Alfonsi E, Montini A, Moglia A, Merlini G, Obici L (2017). Diagnostic challenges in hereditary transthyretin amyloidosis with polyneuropathy: avoiding misdiagnosis of a treatable hereditary neuropathy. J Neurol Neurosurg Psychiatry.

[26] Karam C, Dimitrova D, Heitner SB (2019). Misdiagnosis of hATTR amyloidosis: a single US site experience. Amyloid.

[27] Gonzalez-Lopez E, Gallego-Delgado M, Guzzo-Merello G, de Haro-Del Moral FJ, Cobo-Marcos M, Robles C, Bornstein B, Salas C, Lara-Pezzi E, Alonso-Pulpon L, Garcia-Pavia P (2015). Wild-type transthyretin amyloidosis as a cause of heart failure with preserved ejection fraction. Eur Heart J.

[28] Maurer MS, Bokhari S, Damy T, Dorbala S, Drachman BM, Fontana M, Grogan M, Kristen AV, Lousada I, Nativi-Nicolau J, Cristina Quarta C, Rapezzi C, Ruberg FL, Witteles R, Merlini G (2019). Expert consensus recommendations for the suspicion and diagnosis of transthyretin cardiac amyloidosis. Circ Heart Fail.

[29] Narotsky DL, Castano A, Weinsaft JW, Bokhari S, Maurer MS (2016). Wild-type transthyretin cardiac amyloidosis: novel insights from advanced imaging. Can J Cardiol.

[30] Gertz M, Adams D, Ando Y, Beirão JM, Bokhari S, Coelho T, Comenzo RL, Damy T, Dorbala S, Drachman BM, Fontana M, Gillmore JD, Grogan M, Hawkins PN, Lousada I, Kristen AV, Ruberg FL, Suhr OB, Maurer MS, Nativi-Nicolau J, Quarta CC, Rapezzi C, Witteles R, Merlini G (2020). Avoiding misdiagnosis: expert consensus recommendations for the suspicion and diagnosis of transthyretin amyloidosis for the general practitioner. BMC Fam Pract.

[31] Lousada I, Maurer M, Warner M, Guthrie S, Hsu K, M. G,  (2017). Amyloidosis research consortium cardiac amyloidosis survey: results from patients with ATTR amyloidosis and their caregivers. Orphanet J Rare Dis.

[32] Damy T, Costes B, Hagege AA, Donal E, Eicher JC, Slama M, Guellich A, Rappeneau S, Gueffet JP, Logeart D, Plante-Bordeneuve V, Bouvaist H, Huttin O, Mulak G, Dubois-Rande JL, Goossens M, Canoui-Poitrine F, Buxbaum JN (2016). Prevalence and clinical phenotype of hereditary transthyretin amyloid cardiomyopathy in patients with increased left ventricular wall thickness. Eur Heart J.

[33] Ruberg FL, Maurer MS, Judge DP, Zeldenrust S, Skinner M, Kim AY, Falk RH, Cheung KN, Patel AR, Pano A, Packman J, Grogan DR (2012). Prospective evaluation of the morbidity and mortality of wild-type and V122I mutant transthyretin amyloid cardiomyopathy: the Transthyretin Amyloidosis Cardiac Study (TRACS). Am Heart J.

[34] Benson MD, Teague SD, Kovacs R, Feigenbaum H, Jung J, Kincaid JC (2011). Rate of progression of transthyretin amyloidosis. Am J Cardiol.

[35] Brunjes DL, Castano A, Clemons A, Rubin J, Maurer MS (2016). Transthyretin cardiac amyloidosis in older Americans. J Card Fail.

[36] Castano A, Drachman BM, Judge D, Maurer MS (2015). Natural history and therapy of TTR-cardiac amyloidosis: emerging disease-modifying therapies from organ transplantation to stabilizer and silencer drugs. Heart Fail Rev.

[37] Connors LH, Sam F, Skinner M, Salinaro F, Sun F, Ruberg FL, Berk JL, Seldin DC (2016). Heart failure resulting from age-related cardiac amyloid disease associated with wild-type transthyretin: a prospective, observational cohort study. Circulation.

[38] Mohammed SF, Mirzoyev SA, Edwards WD, Dogan A, Grogan DR, Dunlay SM, Roger VL, Gertz MA, Dispenzieri A, Zeldenrust SR, Redfield MM (2014). Left ventricular amyloid deposition in patients with heart failure and preserved ejection fraction. JACC Heart Fail.

[39] Witteles RM, Bokhari S, Damy T, Elliott PM, Falk RH, Fine NM, Gospodinova M, Obici L, Rapezzi C, Garcia-Pavia P (2019). Screening for transthyretin amyloid cardiomyopathy in everyday practice. JACC Heart Fail.

[40] Lozeron P, Mariani LL, Dodet P, Beaudonnet G, Theaudin M, Adam C, Arnulf B, Adams D (2018). Transthyretin amyloid polyneuropathies mimicking a demyelinating polyneuropathy. Neurology.

[41] Dyck PJ, Lambert EH (1969). Dissociated sensation in amylidosis. Compound action potential, quantitative histologic and teased-fiber, and electron microscopic studies of sural nerve biopsies. Arch Neurol.

[42] Kim DH, Zeldenrust SR, Low PA, Dyck PJ (2009). Quantitative sensation and autonomic test abnormalities in transthyretin amyloidosis polyneuropathy. Muscle Nerve.

[43] Coelho T, Vinik A, Vinik EJ, Tripp T, Packman J, Grogan DR (2017). Clinical measures in transthyretin familial amyloid polyneuropathy. Muscle Nerve.

[44] Carr AS, Pelayo-Negro AL, Evans MR, Laura M, Blake J, Stancanelli C, Iodice V, Wechalekar AD, Whelan CJ, Gillmore JD, Hawkins PN, Reilly MM (2016). A study of the neuropathy associated with transthyretin amyloidosis (ATTR) in the UK. J Neurol Neurosurg Psychiatry.

[45] Adams D (2013). Recent advances in the treatment of familial amyloid polyneuropathy. Ther Adv Neurol Disord.

[46] Gonzalez-Duarte A (2019). Autonomic involvement in hereditary transthyretin amyloidosis (hATTR amyloidosis). Clin Auton Res.

[47] Benson MD, Kincaid JC (2007). The molecular biology and clinical features of amyloid neuropathy. Muscle Nerve.

[48] Abramov D, Weimer LH, Marboe CC, Shimbo D, King DL, Maurer MS (2009). Absence of heart failure in severe cardiac and autonomic amyloidosis: the essential role of sympathetic activation and venous tone in the development of the congestive heart failure syndrome. Congest Heart Fail.

[49] Wixner J, Mundayat R, Karayal ON, Anan I, Karling P, Suhr OB, investigators T,  (2014). THAOS: gastrointestinal manifestations of transthyretin amyloidosis - common complications of a rare disease. Orphanet J Rare Dis.

[50] Mazzeo A, Russo M, Di Bella G, Minutoli F, Stancanelli C, Gentile L, Baldari S, Carerj S, Toscano A, Vita G (2015). Transthyretin-related familial amyloid polyneuropathy (TTR-FAP): a single-center experience in Sicily, an Italian endemic area. J Neuromuscul Dis.

[51] Papoutsidakis N, Miller EJ, Rodonski A, Jacoby D (2018). Time course of common clinical manifestations in patients with transthyretin cardiac amyloidosis: delay from symptom onset to diagnosis. J Card Fail.

[52] Sekijima Y, Uchiyama S, Tojo K, Sano K, Shimizu Y, Imaeda T, Hoshii Y, Kato H, Ikeda S (2011). High prevalence of wild-type transthyretin deposition in patients with idiopathic carpal tunnel syndrome: a common cause of carpal tunnel syndrome in the elderly. Hum Pathol.

[53] Sueyoshi T, Ueda M, Jono H, Irie H, Sei A, Ide J, Ando Y, Mizuta H (2011). Wild-type transthyretin-derived amyloidosis in various ligaments and tendons. Hum Pathol.

[54] Pinney JH, Whelan CJ, Petrie A, Dungu J, Banypersad SM, Sattianayagam P, Wechalekar A, Gibbs SD, Venner CP, Wassef N, McCarthy CA, Gilbertson JA, Rowczenio D, Hawkins PN, Gillmore JD, Lachmann HJ (2013). Senile systemic amyloidosis: clinical features at presentation and outcome. J Am Heart Assoc.

[55] Yanagisawa A, Ueda M, Sueyoshi T, Okada T, Fujimoto T, Ogi Y, Kitagawa K, Tasaki M, Misumi Y, Oshima T, Jono H, Obayashi K, Hirakawa K, Uchida H, Westermark P, Ando Y, Mizuta H (2015). Amyloid deposits derived from transthyretin in the ligamentum flavum as related to lumbar spinal canal stenosis. Mod Pathol.

[56] Geller HI, Singh A, Alexander KM, Mirto TM, Falk RH (2017). Association between ruptured distal biceps tendon and wild-type transthyretin cardiac amyloidosis. JAMA.

[57] Rubin J, Alvarez J, Teruya S, Castano A, Lehman RA, Weidenbaum M, Geller JA, Helmke S, Maurer MS (2017). Hip and knee arthroplasty are common among patients with transthyretin cardiac amyloidosis, occurring years before cardiac amyloid diagnosis: can we identify affected patients earlier?. Amyloid.

[58] Ruberg FL, Grogan M, Hanna M, Kelly JW, Maurer MS (2019). Transthyretin amyloid cardiomyopathy: JACC state-of-the-art review. J Am Coll Cardiol.

[59] Kittleson MM, Maurer MS, Ambardekar AV, Bullock-Palmer RP, Chang PP, Eisen HJ, Nair AP, Nativi-Nicolau J, Ruberg FL (2020). Cardiac amyloidosis: evolving diagnosis and management: a scientific statement from the American Heart Association. Circulation.

[60] Senapati A, Sperry BW, Grodin JL, Kusunose K, Thavendiranathan P, Jaber W, Collier P, Hanna M, Popovic ZB, Phelan D (2016). Prognostic implication of relative regional strain ratio in cardiac amyloidosis. Heart.

[61] Koike H, Tanaka F, Hashimoto R, Tomita M, Kawagashira Y, Iijima M, Fujitake J, Kawanami T, Kato T, Yamamoto M, Sobue G (2012). Natural history of transthyretin Val30Met familial amyloid polyneuropathy: analysis of late-onset cases from non-endemic areas. J Neurol Neurosurg Psychiatry.

[62] Yarlas A, Gertz MA, Dasgupta NR, Obici L, Pollock M, Ackermann EJ, Lovley A, Kessler AS, Patel PA, White MK, Guthrie SD (2019). Burden of hereditary transthyretin amyloidosis on quality of life. Muscle Nerve.

[63] Callaghan BC, Cheng HT, Stables CL, Smith AL, Feldman EL (2012). Diabetic neuropathy: clinical manifestations and current treatments. Lancet Neurol.

[64] Yang H, Sloan G, Ye Y, Wang S, Duan B, Tesfaye S, Gao L (2020). New perspective in diabetic neuropathy: from the periphery to the brain, a call for early detection, and precision medicine. Front Endocrinol (Lausanne).

